# Polarized ATR‐FTIR Spectroscopy Reveals Light‐Induced Peptide Insertion of LAH4 Into Photoswitchable Membranes

**DOI:** 10.1002/cphc.70554

**Published:** 2026-08-31

**Authors:** Celine Eckert, Selina Mertens, Karin Hauser

**Affiliations:** ^1^ Department of Chemistry University of Konstanz Konstanz Germany

**Keywords:** azoPC, LAH4, peptide orientation, photoswitchable membranes, polarized ATR‐FTIR spectroscopy

## Abstract

Photoswitchable biomimetic membranes can be controlled by light and offer the possibility to study the role of membrane dynamics for the interaction with antimicrobial peptides. The α‐helical peptide LAH4, a well‐studied model system, interacts with membranes in a pH‐dependent manner. It binds to the membrane surface at acidic pH and inserts into the membrane at basic pH conditions. Polarized attenuated total reflection (ATR) Fourier‐transform infrared (FTIR) spectroscopy was employed to probe peptide orientation and structural changes, with particular emphasis on the pH‐dependent response of LAH4 to light‐induced membrane perturbations. Photoswitchable azoPC lipids were incorporated in large unilamellar vesicles (LUVs) as well as in DIBMA‐stabilized nanodiscs, and the light‐induced *cis*‐to‐*trans* isomerization of the azobenzene was monitored by characteristic vibrational modes showing a reversible transition between two membrane states for both membrane systems. The measurements reveal that photoswitching between the membrane states induces the reorientation of the surface‐bound peptide toward a membrane‐inserted state. The findings underline the potential of photoswitchable membranes for the study of lipid‐peptide interactions as well as the importance of membrane dynamics for antimicrobial activity.

## Graphical abstract

Photoswitchable membranes mimic membrane dynamics and can be controlled by light. Lipids with an azobenzene moiety undergo a light‐induced isomerization, thereby changing the membrane state. Peptide reorientation and insertion upon membrane photoswitching was observed by polarized ATR‐FTIR spectroscopy highlighting the importance of membrane dynamics for peptide–lipid interaction.

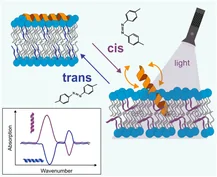

## 1 Introduction

Membrane–protein interactions play a crucial role in a wide range of biological processes, including signal transduction, molecular transport, and cellular communication. A detailed understanding of these processes is essential for elucidating the structural organization and functional dynamics of biological membranes [1]. To investigate such systems under controlled conditions, biomimetic membranes are widely employed. These artificially constructed membrane models mimic the structure and key properties of biological cell membranes. Among them, large unilamellar vesicles (LUVs) are commonly used to study membrane‐related processes due to their well‐defined composition and experimental versatility [2]. In addition to vesicular systems, nanodiscs have emerged as powerful biomimetic membranes for the structural and functional characterization of membrane proteins under physiologically relevant conditions. Nanodiscs consist of a phospholipid bilayer arranged in a disc‐like structure stabilized by membrane scaffold proteins (MSPs) [3, 4, 5] or amphiphilic copolymers. Among the latter, the copolymer diisobutylene/maleic acid (DIBMA) offers several advantages over conventional styrene‐based copolymers such as styrene/maleic acid (SMA). Owing to its aliphatic side chains, DIBMA does not introduce aromatic spectral interferences in spectroscopic studies, exerts only minor effects on lipid chain order, and exhibits enhanced tolerance toward divalent cations [6, 7]. DIBMA‐based nanodiscs therefore provide well‐defined planar membrane systems with tunable dimensions and membrane properties, while maintaining access to both bilayer leaflets. Consequently, nanodiscs have become valuable tools for investigating membrane structure, dynamics, and protein–membrane interactions [8].

**FIGURE 1:**
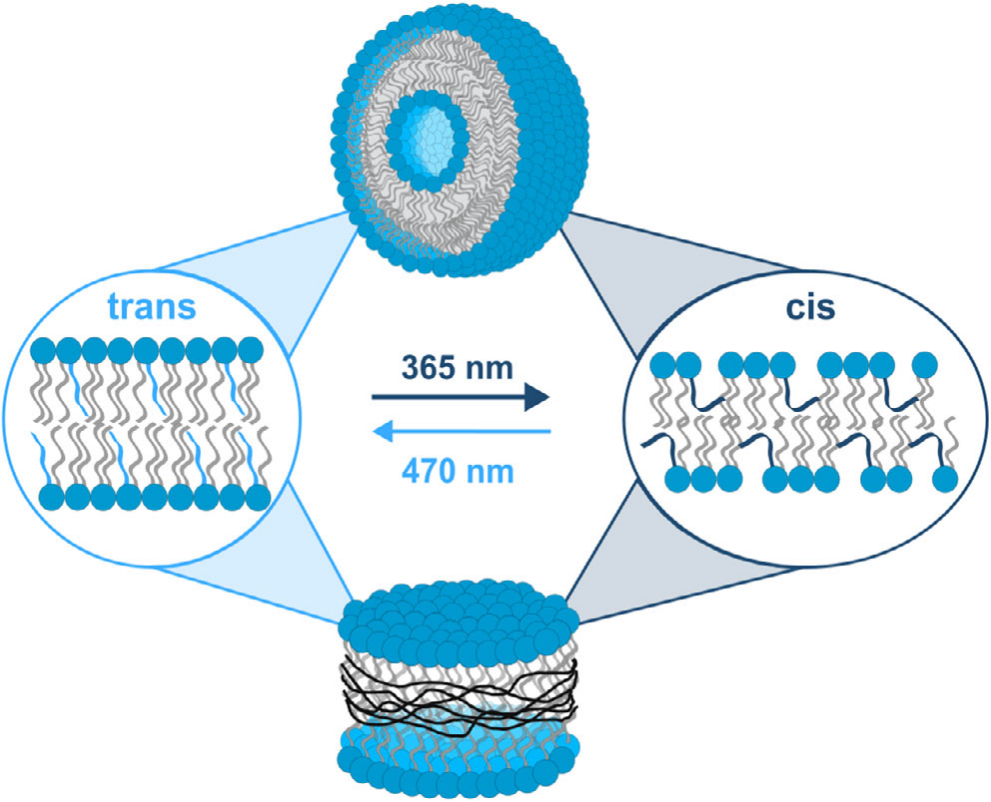
Photoswitchable membrane systems used in this study: vesicles (top) and copolymer‐stabilized nanodiscs (bottom). The lipid bilayer, presented as a zoom‐in, contains photoswitchable lipids (azoPC) that adopt the *trans* configuration (light blue lipid tails) upon irradiation with blue light (470 nm) and the *cis* configuration (dark blue lipid tails) upon irradiation with UV light (365 nm).

Changes in membrane structure and dynamics can alter lipid–peptide interactions and may consequently affect peptide orientation or insertion. This is particularly relevant for antimicrobial peptides (AMPs), whose biological activity often depends on their ability to interact with and insert into lipid bilayers [9, 10]. Therefore, controlled modulation of membrane properties represents an important approach for investigating membrane dynamics and interactions. Light serves as a noncontact, selective, and precise trigger for this purpose. By incorporating the artificial photoswitchable lipid azoPC, membrane properties can be modulated through photoisomerization [11, 12]. AzoPC is a phospholipid containing an azobenzene moiety, which enables reversible photoswitching between its two isomeric states [13, 14]. The thermodynamically more stable *trans*‐azoPC is converted to the *cis* form upon illumination with UV light, while the reverse *cis*‐to‐*trans* isomerization occurs under blue light [11, 14, 15, 16]. This photoswitching process influences key membrane properties, including fluidity, bending rigidity, lateral pressure, and lipid chain organization within the bilayer [11, 13, 17, 18, 19, 20]. At the molecular level, the *trans* state of azoPC is associated with a more ordered membrane structure and an increased bilayer thickness than the *cis* state [17]. The *cis* isomerization of the azoPC lipids leads to an increased lateral area per lipid; the membrane becomes more expanded and is less tightly packed (Figure 1). These light‐induced changes in membrane structure raise the possibility that photocontrol can be used to influence peptide–membrane interactions and potentially trigger changes in peptide orientation or membrane insertion.

The peptide LAH4 is a synthetically designed amphipathic peptide composed predominantly of leucine and alanine residues and contains four histidines [21]. It belongs to the class of AMPs, a group of cationic peptides that contribute to host defense against microbial infections [10, 22]. To exert their biological activity, AMPs typically interact with cellular membranes, specifically through membrane binding, insertion, and permeabilization processes [9, 23]. Upon interaction with lipid membranes, LAH4 adopts an α‐helical structure in contact with lipid membranes in which the four histidine residues are aligned along the upper face of the helix. Owing to the protonation state of these histidines, the orientation of the LAH4 helix with respect to the membrane can be controlled in a pH‐dependent manner [21, 24, 25, 26]. Under acidic conditions, the peptide adopts a predominantly surface‐bound orientation parallel to the membrane plane. At neutral or basic conditions, deprotonation of the histidine residues promotes membrane insertion, resulting in a transmembrane orientation [21, 26, 27, 28]. These pH‐dependent orientational transitions of LAH4 have been extensively characterized by techniques such as solid‐state NMR [21, 24, 26, 27, 29] or polarized attenuated total reflection Fourier‐transform infrared (ATR‐FTIR) spectroscopy [25, 28, 30]. ATR‐FTIR spectroscopy is a powerful method for studying membrane–protein interactions. It enables in situ analysis of protein or membrane characteristics and provides detailed insights into their secondary structure, conformational changes, and dynamics. In particular, difference spectroscopy allows to identify changes in these systems triggered by modifying the environment. Using polarized ATR‐FTIR spectroscopy enhances the sensitivity of molecular orientation, thereby enabling a more detailed characterization of membrane‐associated proteins [25, 28].

In this study, polarized ATR‐FTIR spectroscopy was employed to investigate the model peptide LAH4 in photoswitchable membrane systems, large unilamellar vesicles and DIBMA‐stabilized nanodiscs. The photoswitching dynamics of both membrane systems were characterized by time‐resolved measurements. Building on these experiments, the central objective was to determine whether light‐induced membrane perturbations induce lipid–peptide interactions and potentially trigger changes in peptide conformation. More specifically, this work addresses the question of whether photoswitching‐induced changes in membrane structure and dynamics can affect the membrane insertion process of an AMP. To investigate this possibility, the orientational response of LAH4 to the photoswitching of the membrane was analyzed by polarized ATR‐FTIR spectroscopy under pH‐dependent conditions.

## 2 Materials and Methods

### 2.1 Sample Preparation

1 mg of LAH4 (KKALLALALHHLAHLALHLALALKKA) (GenScript, China) was dissolved in a mixture of 600 µL chloroform and 300 µL methanol (2:1, v/v). For the sample preparation, 100 µL of the LAH4 solution was dried in a glass vial under a gentle nitrogen stream and subsequently lyophilized overnight. A phosphate buffer (0.1 M sodium phosphate, 0.15 M sodium chloride) (Thermo Scientific, USA) was prepared and adjusted to pH 4.0 using hydrochloric acid (Merck, Germany) and to pH 7.0 using sodium hydroxide (Merck, Germany). Finally, 35 µL of the respective buffer was added to 0.111 mg LAH4.

Biomimetic membranes were prepared as large unilamellar vesicles as well as nanodiscs using identical lipid compositions. Lipids of POPC (1‐palmitoyl‐2‐oleoyl‐glycero‐3‐phosphocholine, Avanti Polar Lipids, USA) were dissolved in chloroform at a concentration of 25 mg/mL, and photoswitchable azoPC (1‐stearoyl‐2[(E)‐4‐(4‐((4‐butylphenyl)diazinyl)phenyl)butanoyl]sn‐glycero‐3‐phosphocholine, Avanti Polar Lipids, USA) in chloroform at 5 mg/mL. For a pure POPC sample, 1.25 mg of lipids were used with 250 µL of phosphate buffer. To obtain a 1:1 POPC‐to‐azoPC ratio, 25 µL POPC and 125 µL azoPC were mixed in a glass vial, dried under a nitrogen stream, and subsequently lyophilized. The resulting lipid film (1.25 mg) was rehydrated in phosphate buffer (adjusted to the respective pH value) to a final concentration of 5 mg/mL. The suspension was subjected to three 40‐s sonification cycles using a tip sonicator (Hielscher Ultrasound Technology, Germany). LUVs were then generated by extrusion through a 100‐nm pore‐size polycarbonate filter. For both vesicles and nanodiscs, LAH4 was added at a molar lipid‐to‐peptide ratio of 11:1 (MW (POPC) = 759.6 g/mol, MW (1:1 POPC/azoPC mixture) = 794.6 g/mol). The samples were stored at 4 °C for at least 3 h prior to use. The peptide concentrations are quite high and could cause membrane disturbances. However, we have reasonable experimental indications that the membrane integrity is preserved at this peptide concentration in the case of the model peptide LAH4. The dichroic spectra and pH‐dependent helix orientations were highly reproducible, which is not expected for a membrane with defects, as the peptide would distribute more randomly. In addition, we observe stable water bands as indicator for membrane integrity [31]. Nanodiscs were assembled using the copolymer DIBMA (diisobutylene‐maleic acid, anatrace, USA). DIBMA was dissolved in phosphate buffer (adjusted to the respective pH value) at a concentration of 12 mg/mL, mixed thoroughly, and filtered through a 0.22‐µm membrane filter (Roth, Germany). DIBMA was then added to the lipid–peptide mixture (or just the lipid mixture), resulting in a molar lipid‐to‐peptide‐to‐copolymer ratio of 11:1:0.25. The mixture was incubated in a 38 °C water bath for at least 3 h, during which the initially turbid suspension became optically clear, indicating successful nanodisc formation. Rapid‐scan measurements were performed with LUV samples using Tris‐HCl buffer with 50 mM Trizma base (Sigma‐Aldrich, USA) and 100 mM NaCl (Carl Roth, Germany). The pH was adjusted to 7.4 using 1 N sodium hydroxide and 1 N hydrochloric acid (Merck, Germany). To avoid spectral overlap of a Tris‐HCl buffer mode at pH 4.0 with the amide I region, all measurements with LAH4 were finally performed in phosphate buffer.

### 2.2 ATR‐FTIR Spectroscopy

The FTIR spectra were recorded using a Vertex 80v spectrometer (Bruker Optics, Germany) and a Bio‐ATR II cell (Bruker Optics, Germany) equipped with a multireflective silicon crystal. Each spectrum was acquired with 32 scans at a spectral resolution of 4 cm^−1^. For polarized measurements, an infrared polarizer (PIKE Technologies, USA) was employed, whose alignment was controlled remotely. The dichroic spectra were obtained by subtracting the spectra recorded with perpendicular polarization (s‐polarized) from the spectra recorded with parallel polarization (p‐polarized). Because of the difference in the relative power of the evanescent field, the s‐polarized spectra have to be multiplied by the factor *R* ^ATRiso^ = 1.34. This factor has been observed in isotropically oriented samples and is explained in detail in the literature [28, 30]. For the dichroic spectra of the pure POPC membrane, in addition to the factor 1.34, the spectrum at pH 7.0 was scaled by a factor of 2.7 to match the intensity of the ester band (in the absorption spectra). The internal reflection element (IRE) (silicon *n*_1_ = 3.42) was used at an incidence angle of 45°. For a sample with a refractive index of *n*_2_ = 1.55, the resulting penetration depth at 1650 cm^−1^ is 0.47 μm for perpendicular and 0.84 μm for parallel polarized light [30]. During the measurements, the sample chamber was continuously purged with nitrogen, and the ATR cell temperature was maintained at 25 °C using a water bath equipped with an integrated E300 thermostat (LAUDA‐Brinkmann, USA). All spectra were baseline‐corrected using MATLAB software (Mathworks, USA). For all measurements, the IRE of the ATR‐cell was polished with a 0.1‐µm diamond paste. 10 μL of LUVs or 12.8 µL of nanodiscs was deposited onto the crystal and gently dried under a nitrogen stream for 10 min. Thereafter, the water bath for the temperature control of the ATR cell was switched on, and the sample was dried at 35 °C for another 2 h. The drying procedure is required to produce oriented samples for the polarized measurements, in particular for the LUV samples. Spherical vesicles would show all possible orientations for the reconstituted peptide. By drying the vesicles on the crystal, they will mainly form planar multibilayers with the lipids oriented parallel to the surface normal. The drying procedure is also advantageous for the nanodiscs that already have a planar geometry since the drying will enhance the preferred orientation of the lipids parallel to the surface normal. We assume that the bilayer integrity is retained after the drying process. During the measurements, the temperature was maintained at 25 °C. The sample was illuminated under continuous light using LEDs (Ocean Optics, USA) with either blue (470 nm, 418 µW/cm^2^) or UV (365 nm, 1.94 mW/cm^2^) light. Spectra were recorded sequentially under blue and UV illumination, with both parallel and perpendicular polarizations. In total, four illumination cycles were conducted. Time‐resolved measurements were recorded in rapid‐scan mode with double‐sided forward–backward scans, a maximum scanner velocity of 320 kHz, a temporal resolution of 68 ms, and a spectral resolution of 4 cm^−1^. Illumination conditions were set to 3.24 mW/cm^2^ for blue (470 nm) and 15.59 mW/cm^2^ for UV (365 nm) light.

## 3 Results and Discussion

### 3.1 Characterization of pH‐Dependent LAH4 Orientation in LUVs and Nanodiscs

**FIGURE 2:**
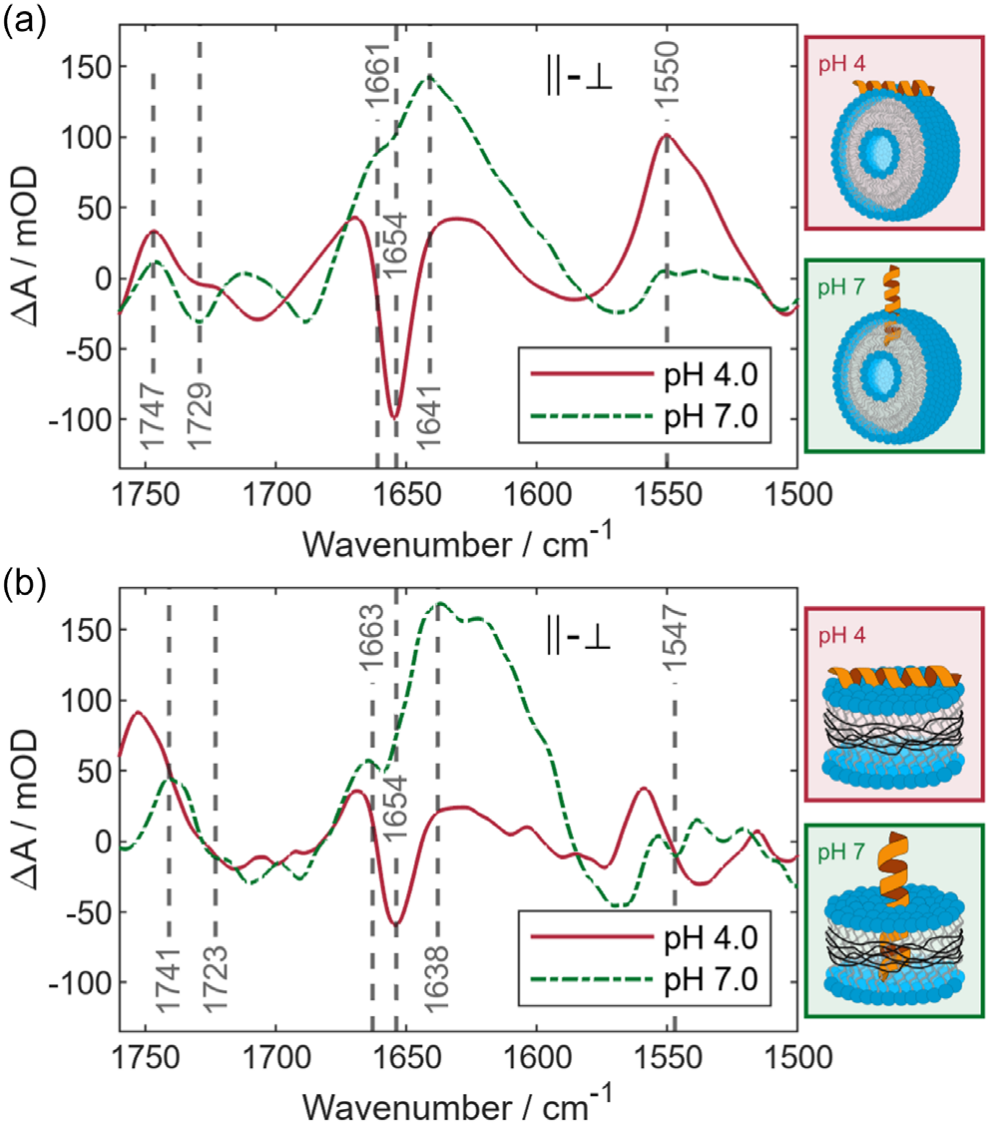
Polarized ATR‐FTIR difference spectra of POPC (a) LUVs and (b) nanodiscs with the peptide LAH4 at pH 4.0 (red, solid) and pH 7.0 (green, dashed). Dichroic spectra were obtained by subtracting the perpendicular from the parallel polarized spectra. Schematic representation of peptide (orange) orientations in LUVs (top) and nanodiscs (bottom) at pH 4.0 (red box) and pH 7.0 (green box). It should be noted that the LUVs will not remain intact after the drying procedure and mainly form planar multibilayers on the IRE surface.

The histidine‐rich amphipathic peptide LAH4 adopts predominantly an α‐helical structure upon interaction with lipid membranes. LAH4 has been designed to change its orientation relative to the membrane in a pH‐dependent manner [21]. This pH‐dependent behavior arises from the protonation and deprotonation of histidine residues, which possess pKa values between 5.4 and 6.0 [21, 28, 30]. The interaction with membranes and antimicrobial activity of LAH4 were extensively analyzed over the last years [26, 32, 33]. Furthermore, it has been demonstrated that polarized ATR‐FTIR spectroscopy is an effective method for characterizing the orientation of LAH4 in orientated multilayer POPC membranes [28, 30]. Here, we have used different biomimetic membrane systems, LUVs and nanodiscs. Figure 2 shows polarized ATR‐FTIR difference spectra of POPC LUVs and POPC nanodiscs containing the peptide LAH4 at pH 4.0 and 7.0, respectively. In general, the absorption spectra are dominated by the amide I and amide II bands, the peptide backbone vibrations providing characteristic information on the secondary structure. Spectra were recorded with parallel and perpendicular polarized light, and dichroic difference spectra (parallel minus perpendicular) were generated that reflect the orientational distribution of the peptide. To account for the differences in the relative power of the evanescent field, it is necessary to multiply the perpendicular spectrum by a correction factor *R* ^ATRiso^ = 1.34, i.e., the dichroic ratio observed for an isotropically oriented sample, as shown previously [28, 34]. Accordingly, the s‐polarized spectra were multiplied by 1.34 before subtracting from the corresponding p‐polarized spectra.

In Figure 2a, the dichroic spectrum of LAH4‐containing LUVs at pH 4.0 (red, solid line) exhibits a pronounced negative band at 1654 cm^−1^ in the amide I region and a distinct positive signal at 1550 cm^−1^ in the amide II region, indicating that the helix axis is predominantly oriented parallel to the membrane surface [28, 30]. In contrast, the spectrum recorded at pH 7.0 (green, dashed line) displays a broad positive amide I region, with a maximum at 1641 cm^−1^ and a shoulder at 1661 cm^−1^. The amide II region around 1550 cm^−1^ does not exhibit a clear dichroism. Nevertheless, the positive amide I dichroism indicates that the helix axis is predominantly oriented perpendicular to the membrane surface, consistent with the membrane insertion of the peptide. Similar observations were reported by Bechinger et al., who identified a positive amide I band and a negative amide II band at pH 9.0 [28, 30]. The close agreement between the dichroic spectra at pH 7.0 and pH 9.0 suggests that the orientational behavior of LAH4 is comparable, indicating no substantial differences between these alkaline conditions. Both spectra in Figure 2a also exhibit characteristic features in the ester stretching vibrations of POPC, appearing as a positive component at 1747 cm^−1^ and a negative component at 1729 cm^−1^, a behavior that has previously been observed with POPC membranes [28, 30].

The same studies were performed with a different membrane system. For this purpose, POPC nanodiscs were assembled with the peptide at pH 4.0 and 7.0, and the resulting dichroic difference spectra are shown in Figure 2b. At pH 4.0, the spectrum exhibits the same dichroism as for the LUVs, a pronounced negative amide I band at 1654 cm^−1^, accompanied by a positive band in the amide II region, revealing the predominantly planar orientation of the peptide on the nanodisc surface. At pH 7.0, a broad positive band is detected in the amide I region with a pronounced peak at 1638 cm^−1^ and a shoulder at 1663 cm^−1^. Notably, this feature is broader than the corresponding band observed in LUVs. One possible explanation is an overlap with the deprotonated carboxylate (COO^−^) vibration of the DIBMA copolymer used to assemble the nanodiscs. At pH 9.0, this copolymer band becomes clearly visible at 1580 cm^−1^ (Figure S1). To minimize spectral interference in the amide I region, all polarized measurements were consequently performed at pH 7.0 rather than at higher pH values. In addition, a small negative band at 1547 cm^−1^ is observed in the amide II region of the dichroic spectrum. Together with the broad positive amide I feature, this spectral pattern is consistent with a predominantly perpendicular orientation of the LAH4 helix axis within the nanodisc membrane. Two bands are observed at 1741 and 1723 cm^−1^ and attributed to the lipid's C=O stretching vibration.

Overall, these results demonstrate that LAH4 adopts distinct orientations at pH 4.0 and pH 7.0 in both membrane systems, LUVs and nanodiscs. Despite the additional spectral contributions from the copolymer, nanodiscs provide a suitable membrane model system for investigating peptide orientation by polarized ATR‐FTIR spectroscopy.

### 3.2 Controlled Generation of Different Membrane States Through Light Switching of azoPC Lipids

**FIGURE 3:**
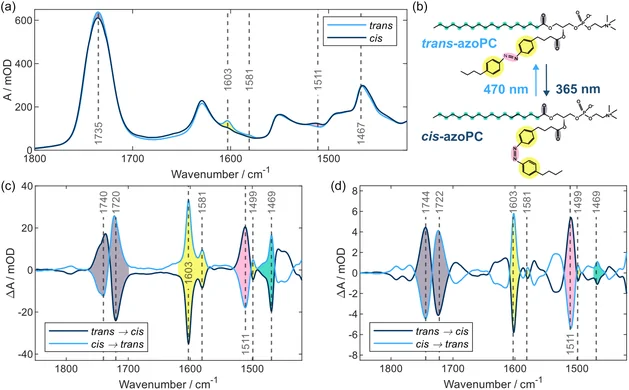
(a) Absorption spectra of LUVs composed of 50% POPC and 50% azoPC at pH 7.4 (Tris‐HCl) in the *trans* (light blue) and *cis* (dark blue) state. (b) Chemical structures of *trans* and *cis* isomerization forms of azoPC induced by illumination with 365 and 470 nm light, respectively. Difference spectra of (c) LUVs and (d) nanodiscs composed of 50% POPC and 50% azoPC at pH 7.4 (Tris‐HCl). The spectra illustrate the photoinduced transition of azoPC from *trans*‐to‐*cis* isomer (dark blue) and from *cis*‐to‐*trans* isomer (light blue). The marker bands and their corresponding structural regions are highlighted in matching colors.

Photoswitchable membranes provide a useful approach for the control of membrane structure and dynamics through illumination with blue or UV light. Incorporation of the photoresponsive lipid azoPC into the membrane enables reversible *cis*–*trans* isomerization, which can be detected through changes in characteristic IR marker bands. The membranes examined are composed of POPC and azoPC. The absorption spectra of LUVs in their respective *trans* or *cis* states are shown in Figure 3a. Differences can be observed in characteristic bands, particularly at 1603 and 1511 cm^−1^, which are associated with the ring breathing modes of azobenzene and N=N stretching vibrations [35]. Additional spectral changes are evident in the ester carbonyl stretching region at 1735 cm^−1^. Figure 3b illustrates the photoinduced isomerization transition of the azoPC lipids upon illumination with blue or UV light. The color‐coded structural elements correspond to the vibrational modes responsible for the observed changes in the absorption spectra.

The changes in the vibrational modes become more obvious in difference spectra (Figure 3c,d) where all lipid vibrational modes are canceled out that do not change upon the photoinduced isomerization reaction. In the *trans‐*to‐*cis* difference spectrum, the *trans* absorption spectrum is subtracted from the *cis* absorption spectrum; therefore, the positive bands represent the *cis* state and the negative bands the *trans* state. It is the opposite for the *cis‐*to‐*trans* difference spectrum, positive bands represent the *trans* state, and the negative bands the *cis* state. The pronounced ester band at 1735 cm^−1^ in the absorption spectra results in a positive and negative band in the difference spectra with contributions of anhydrous C=O stretching at 1740 cm^−1^ and hydrated C=O stretching at 1720 cm^−1^ [35]. This indicates that the ester carbonyl of the azoPC lipid is more hydrated in the *trans* state and less hydrated in the *cis* state as a result of conformational change of the azobenzene. This observation may be explained by the different alignment of the azoPC lipids in the *trans* and *cis* states. In the *trans* state, the polar head groups and nonpolar alkyl chains are more clearly separated, facilitating water penetration into the interfacial region. Upon isomerization to the *cis* state, part of the nonpolar azobenzene moiety is displaced toward the polar head‐group region, which may reduce local hydration and hinder water incorporation near the ester groups. The ester groups of POPC also absorb within this spectral range. However, the absorption changes observed for the photoswitchable lipids are markedly larger [36]. Additional characteristic bands distinguish the two isomers. The *trans* form, associated with azobenzene ring breathing modes, shows positive peaks at 1603 and 1581 cm^−1^. In contrast, the *cis* isomer exhibits peaks at 1511 cm^−1^, corresponding to the N=N stretching vibration, and at 1499 cm^−1^, reflecting modified ring vibrations following isomerization. The 1469 cm^−1^ changes correspond to the CH_2_ vibration of the alkyl chains from POPC and azoPC [35]. All specific assignments of the characteristic vibrational modes are summarized in Table 1. The photoinduced isomerization reaction can be repeated indefinitely in forward and reverse directions [35, 37]. The *trans*‐to‐*cis* difference spectrum (dark blue) and the *cis‐*to‐*trans* difference spectrum (light blue) are mirror images; positive bands become negative and vice versa, revealing the reversible photoswitching, as expected.

**TABLE 1:** Vibrational assignment of the marker bands of azoPC [35].

Wavenumber, cm^−1^	Assignment
1740	C=O stretching anhydrous ester
1720	C=O stretching hydrated ester
1603	Ring breathing mode in *trans* azoPC
1581	Ring breathing mode in *trans* azoPC
1511	N=N stretching in *cis* azoPC
1499	Ring breathing mode in *cis* azoPC
1469	CH_2_ scissoring

The same marker bands observed in the measurements of LUVs (Figure 3c) are also present in the difference spectra of nanodiscs (Figure 3d). This finding indicates that membrane dynamics can also be induced in nanodiscs through photoswitching of the azoPC lipids. However, the magnitude of the absorbance changes is significantly reduced compared to vesicles, likely due to the copolymer that is wrapped around the lipid bilayer in the nanodiscs and constrains the photoinduced conformational changes within the membrane.

**FIGURE 4:**
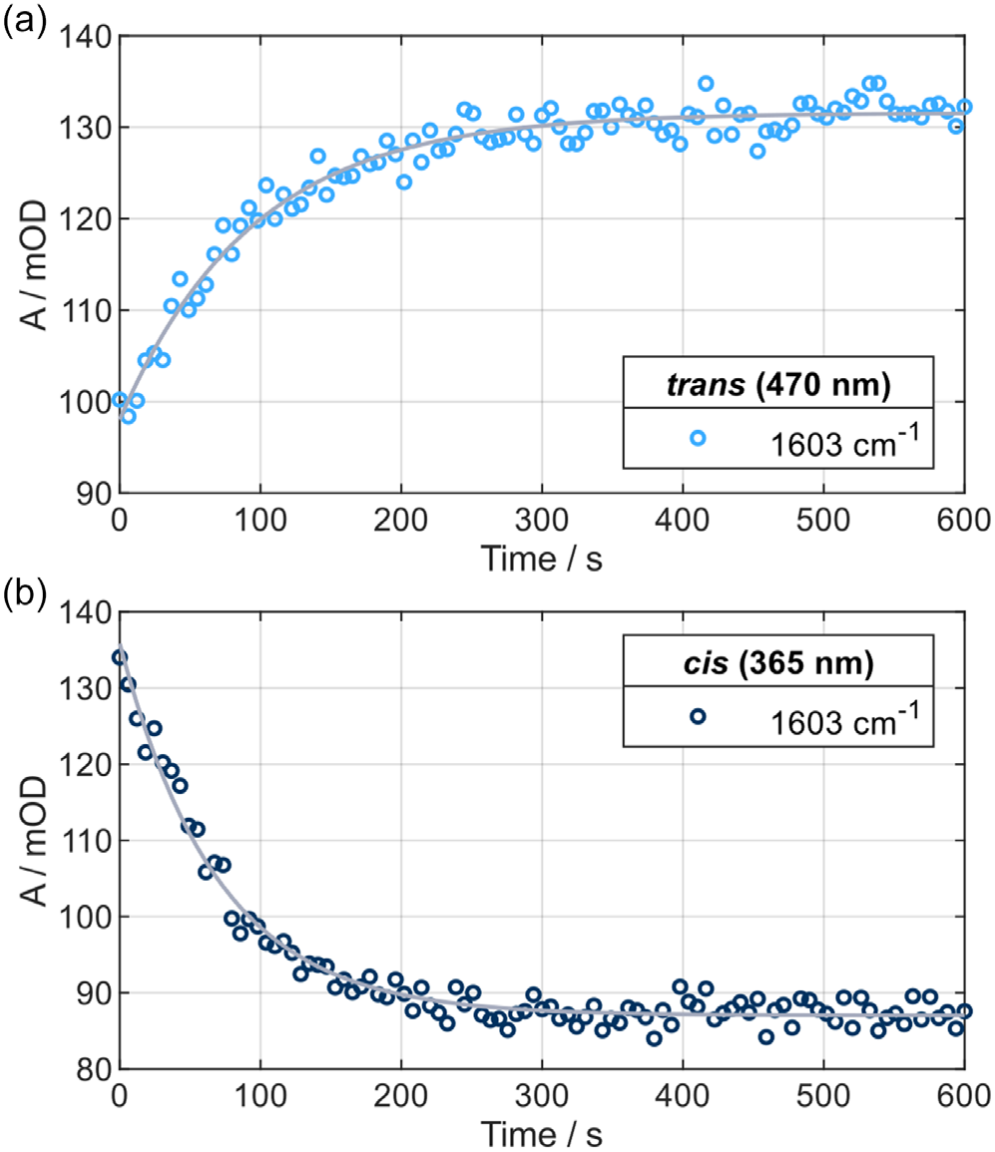
Temporal evolution of the absorbance measured at 1603 cm^−1^ for photoswitchable LUVs 50% POPC and 50% azoPC. Data in (a) were recorded during photoexcitation with 470‐nm light and in (b) during illumination with 365 nm. Both datasets were fitted with a monoexponential function (gray), resulting in time constants of *τ*_*trans*_ = 94 ± 4 s and *τ*_*cis*_ = 69 ± 2 s.

The results demonstrate that photoswitchable lipids are well suited to induce conformational changes within different biomimetic membrane systems and to switch between defined membrane states by light control. We have also studied the dynamics of the membrane switching by time‐resolved rapid‐scan ATR‐FTIR measurements. As discussed above, several characteristic marker bands can be used to characterize the membrane photoswitching. The band at 1603 cm^−1^, assigned to the ring breathing mode of azobenzene, was selected as a representative marker to monitor the switching kinetics and to determine how fast the photostationary membrane state occurs. Figure 4 shows the temporal evolution of this azoPC lipid mode during continuous irradiation at either 365 or 470 nm over a period of 600 s, corresponding to the induction of the respective *cis* and *trans* states. Notably, the two transients exhibit nearly mirror‐image behavior. For both cases, the signal approaches a plateau after approximately 350 s, indicating the establishment of a photostationary state. This observation suggests that the isomerization process within the membrane is largely complete after about 350 s, irrespective of the irradiation wavelength.

The extracted time constants provide further insight into the kinetics of the switching process. A time constant of 94 ± 4 s was obtained for the *cis*‐to‐*trans* isomerization, whereas the *trans*‐to‐*cis* isomerization exhibited a time constant of 69 ± 2 s. These results indicate that conversion to the *cis* state proceeds more rapidly than conversion to the *trans* state under the investigated conditions. It should be noted, however, that the observed time constants depend on factors such as illumination intensity and other experimental parameters [37, 38]. Overall, the data show that an illumination period of approximately 5 min is sufficient to drive the membrane close to its photostationary state and thus achieve efficient photoswitching. The reversibility of the photoswitching process under alternating blue and UV light illumination was confirmed for at least 20 cycles (Figure S2).

### 3.3 LAH4 Peptide Interaction With Photoswitched Membrane States

The incorporation of photoswitchable lipids introduces controlled dynamics within the membrane. In the presence of the peptide LAH4, these membrane perturbations may affect lipid–peptide interactions, which are expected to differ between pH 4.0 and pH 7.0 due to the distinct orientations adopted by the peptide under these conditions. If different membrane states are induced by photoswitching, the resulting structural changes within the bilayer may impact the orientation of LAH4. Thus, we prepared azoPC/POPC membranes with LAH4 at pH 4.0 and pH 7.0 and analyzed the effect of photoswitching on the peptide. We expect the characteristic azoPC marker bands in the difference spectra showing the photoswitching of the membrane as in Figure 3, but in addition, we get information on the peptide conformation in the amide I region as well as potential frequency shifts in the lipid bands due to peptide–lipid interaction. One could speculate that the azobenzene switch interacts more with the peptide at pH 7.0 when the peptide is inserted into the membrane than at pH 4.0 when the peptide is bound to the membrane surface and is not in contact with the azobenzene moiety.

**FIGURE 5:**
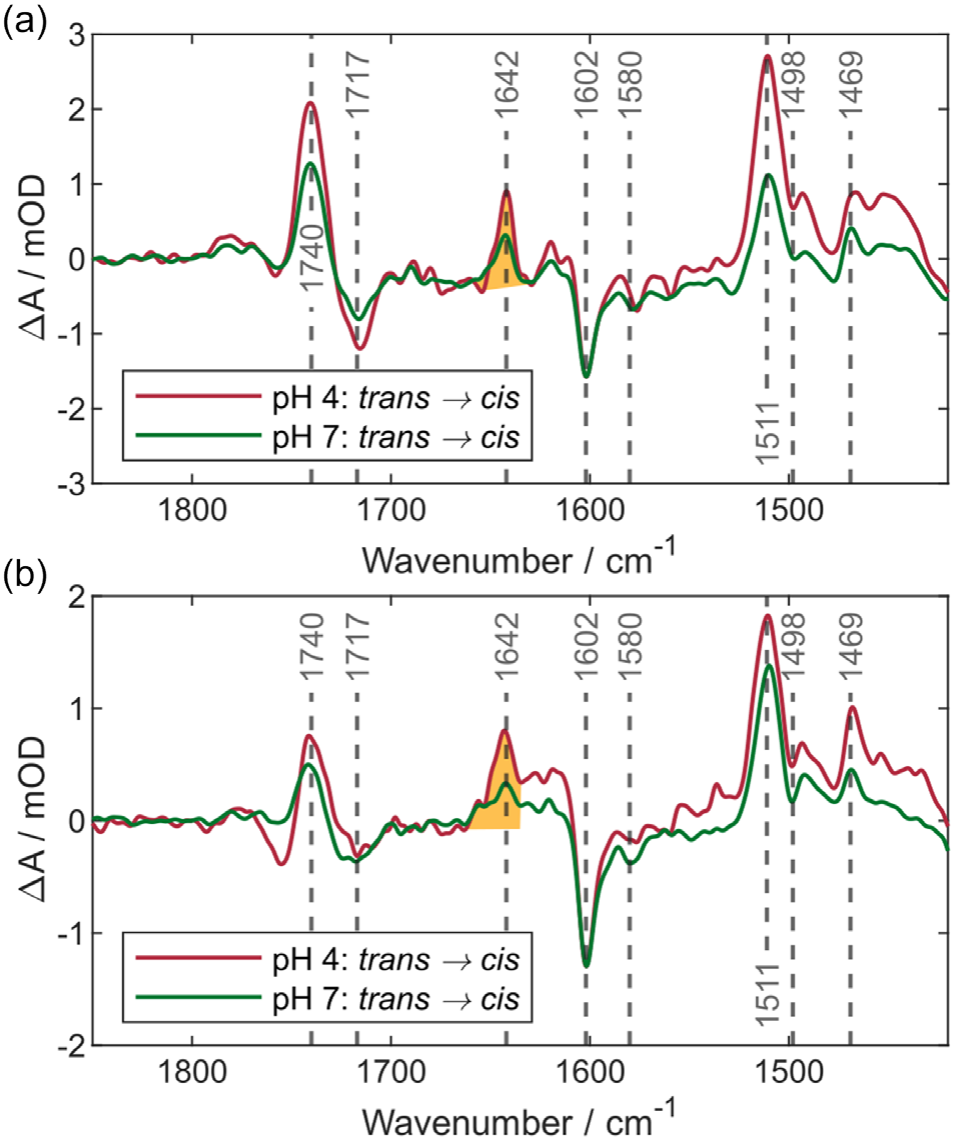
ATR‐FTIR difference spectra of (a) LUVs and (b) nanodiscs composed of 50% POPC and 50% azoPC with the peptide LAH4 at pH 4.0 (red) and pH 7.0 (green). Structural changes of the peptide photoinduced by the *trans*‐to‐*cis* transition of the membrane occur in the amide I region marked in orange.

Measurements with LUVs (Figure 5a) and nanodiscs (Figure 5b) containing LAH4 reveal if there are differences dependent on the pH, LAH4 conformation, and interaction with the membrane. For clarity, only the spectra corresponding to the *trans*‐to‐*cis* membrane transition are shown. The spectra were scaled to the azobenzene band intensity at 1602 cm^−1^ to facilitate comparison between the two pH values, in particular in the amide I region where conformational changes of the peptide become obvious if they are induced by the membrane switching. The mirror‐imaged difference spectra for the reverse *cis*‐to‐*trans* transition show the same results, as expected, and are provided in Figure S3. In Figure 5a, the LUV spectra exhibit the characteristic marker bands associated with the formation of the *cis* isomer of the azoPC lipid as in Figure 3c. There are no significant shifts observed in the lipid bands, indicating that the photoswitching of the membrane states is similar as without the peptide and thus not changed significantly by peptide–lipid interactions, neither at pH 4.0 nor at pH 7.0. However, we clearly observe an effect of the membrane photoswitching on the peptide conformation by the spectral changes in the amide I region at 1642 cm^−1^ for both pH values, thus when the peptide is predominantly bound to the membrane surface as well as when it is inserted. If the membrane photoswitching would have no effect on the peptide conformation, no spectral changes in the amide I region would have been observed because the same amide I absorbance in the *trans* and the *cis* state of the membrane would cancel out in the difference spectrum.

Similar observations were obtained for the nanodiscs (Figure 5b). For better comparison of the two pH values, the spectra were also scaled to the same intensity of the azobenzene band at 1602 cm^−1^. The characteristic marker bands of azoPC showing the membrane photoswitching become visible and are not significantly shifted compared to the membrane without the peptide (Figure 3d) as it was also the case for the LUV membranes. Again, the intensities of the bands are smaller for the nanodiscs compared to the LUV membranes as the scaffold copolymer constrains the conformational changes within the nanodisc. Clearly visible is a pronounced amide I band observed at 1642 cm^−1^ for both pH values. The intensity of the amide I band is stronger at pH 4.0 than at 7.0 as it was already observed for the LUV membranes. This may indicate that photowitching‐induced membrane perturbations have a stronger effect on lipid–peptide interactions when LAH4 is predominantly bound to the membrane surface.

### 3.4 Photoswitched Membrane Dynamics Induces Peptide Reorientation

Since unpolarized measurements have revealed photoswitching‐dependent changes in the amide region of LAH4, indicating that the peptide is affected by membrane dynamics, we performed polarized ATR‐FTIR measurements. Polarized ATR‐FTIR measurements reveal the orientation of LAH4 with the membrane as we have shown with nonswitchable POPC LUVs and nanodiscs (Figure 2). Under acidic conditions, the α‐helical peptide adopts a predominantly surface‐bound orientation, whereas at neutral to basic pH, the α‐helix inserts into the membrane. The photoswitching of the membrane generates changes in membrane structure and dynamics. These changes affect the membrane properties such as fluidity, chain order, bending rigidity, and lateral pressure [17, 38, 39]. At the molecular level, the *trans* state of azoPC is associated with a more ordered membrane structure and an increased thickness, whereas the *cis* state leads to a less ordered structure and a reduction in membrane thickness [17]. Consequently, photoswitching alters the physical environment surrounding the peptide and may therefore influence its orientation and membrane interactions.

**FIGURE 6:**
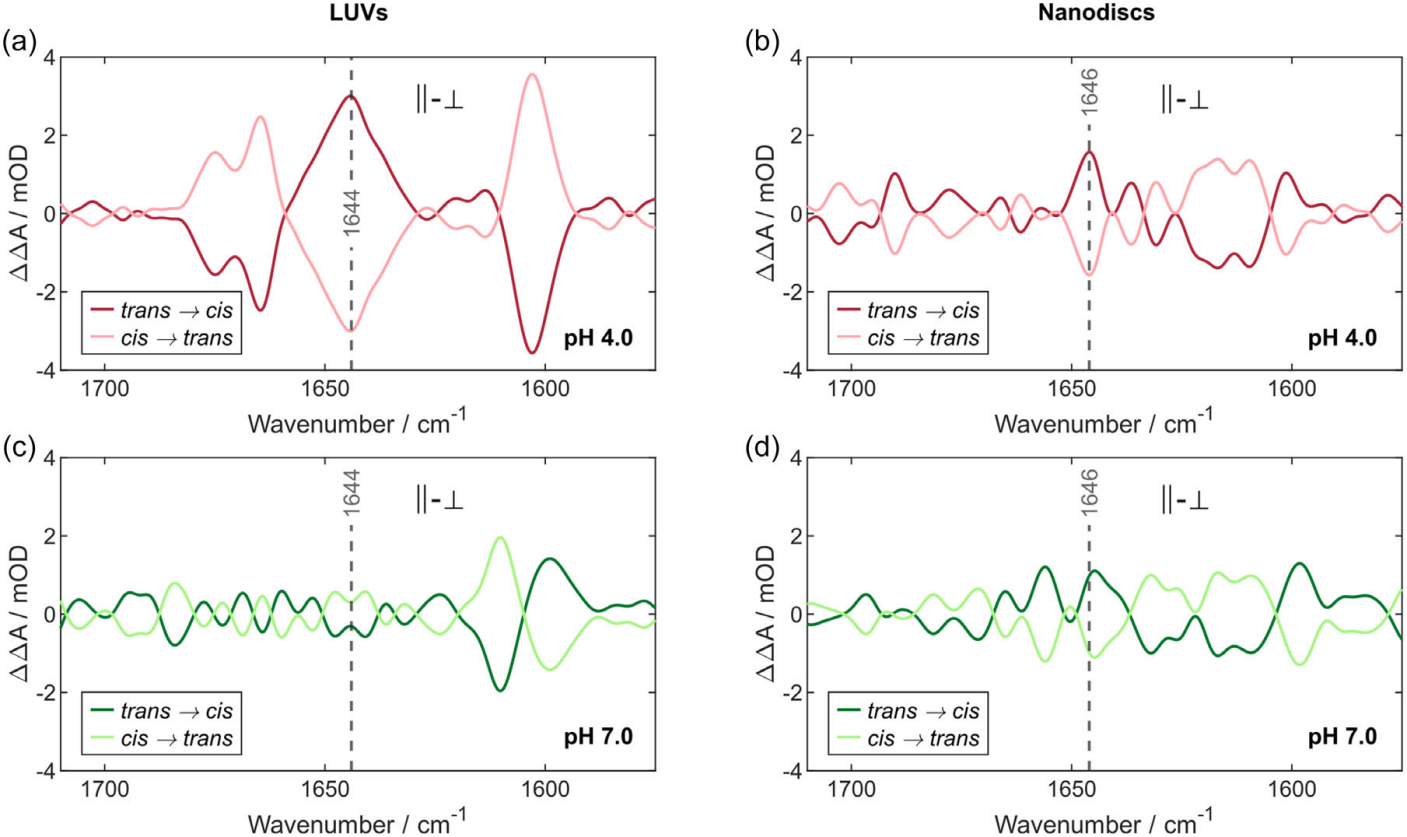
Polarized ATR‐FTIR double difference spectra of (a,c) LUVs and (b,d) nanodiscs composed of 50% POPC and 50% azoPC with the peptide LAH4 at pH 4.0 (a, b, red) and pH 7.0 (c, d, green) for both photoisomerization transitions of azoPC (*trans* ‐to‐*cis* and *cis‐*to*‐trans*). The dichroic spectra reveal the effect of membrane photoswitching on the orientation of the LAH4 helix. The transition to the *cis* state is accompanied by a positive amide I band of the peptide at 1644 cm^−1^ at pH 4.0. A positive dichroic amide I band indicates the membrane insertion of LAH4; thus, the photoinduced change in the membrane state results in a partial reorientation of the surface‐bound LAH4 toward membrane insertion. Switching back to the *trans* state shows a mirror‐imaged spectrum with a negative amide I band, revealing that the peptide returns back to the initial surface‐bound orientation. At pH 7.0, there are no significant amide I changes observable, indicating that the photoinduced change in the membrane state does not affect the orientation of the inserted peptide.

Figure 6 shows polarized ATR‐FTIR double difference spectra of LUVs and nanodiscs containing azoPC and LAH4 at the different pH values upon photoswitching between the *cis* and *trans* membrane states. The spectra are presented separately for the *cis* and *trans* states of the membrane. To generate these double difference spectra, the spectra recorded in the *cis* and *trans* states were first subtracted from one another for each polarization direction separately. Thus, a photoswitching‐induced difference spectrum was obtained for both the p‐polarized and s‐polarized measurements. Subsequently, the s‐polarized difference spectra were multiplied by a factor of 1.34 and subtracted from the corresponding p‐polarized difference spectra to obtain the final dichroic double‐difference spectra. In this approach, the first subtraction isolates spectral changes induced by photoswitching, whereas the second subtraction extracts the orientational information contained in the polarized spectra. Therefore, the changes in the amide I region reflect orientational changes of the peptide if they are induced by the photoswitching between the two different membrane states.

**FIGURE 7:**
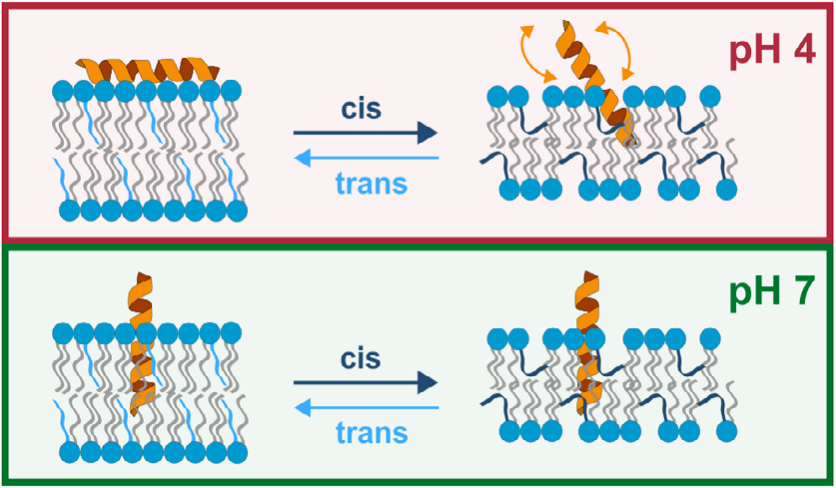
Schematic representation of the lipid bilayer in the *trans* (light blue, left) or *cis* (dark blue, right) state and orientational changes of LAH4 after photoinduced transition. The α‐helical peptide (orange) adopts a surface‐bound orientation at pH 4 (red box) in the *trans* state but reorients and partially inserts into the membrane in the *cis* state due to increased bilayer expansion and membrane “opening”. At pH 7 (green box), the peptide is inserted into the membrane in both the *trans* and the *cis* state, and the photoswitching of the membrane states does not induce an orientational change of the peptide.

As shown in Figure 2, LAH4 adopts a predominantly surface‐bound orientation at pH 4.0, which is characterized by a negative amide I band in the dichroic spectra. Consistent with this behavior, the LUV spectrum corresponding to the *cis*‐to‐*trans* isomerization in Figure 6a exhibits a negative amide I band. This indicates that the peptide remains oriented parallel to the membrane surface in the *trans* state, which represents the thermodynamically more stable membrane configuration. In contrast, the spectrum associated with the *trans*‐to‐*cis* isomerization displays a positive amide I band at 1644 cm^−1^. Based on the orientational assignments established in Figure 2, this spectral pattern is consistent with a more inserted orientation of LAH4 within the membrane. These observations suggest that, while the peptide adopts its expected surface‐bound orientation in the *trans* state at pH 4.0, the structural changes accompanying photoswitching to the *cis* state alter the membrane environment sufficiently to affect peptide orientation. As the *cis* state is associated with an increased lateral area per lipid and a reduced bilayer thickness, the membrane becomes more expanded and less tightly packed [17]. Under these conditions, LAH4 may partially reorient and penetrate more deeply into the membrane. A schematic illustration of this process is presented in Figure 7.

A similar trend is observed for the nanodiscs (Figure 6b). At pH 4.0, the spectrum associated with the *trans* state exhibits a negative amide I band, whereas the corresponding spectrum for the *cis* state displays a positive feature in the amide I region. This behavior is consistent with the observations made for LUVs. It must be noted, however, that these spectral changes are relatively small. As described above, the photoswitching between the *trans* and *cis* states is less effective in nanodiscs (Figure 3d) than in LUVs (Figure 3c), resulting in smaller signal intensities. Therefore, the photoinduced conformational changes and amide signals are also smaller in the spectra of nanodiscs. As a result, the signal‐to‐noise ratio becomes a limiting factor in the interpretation of these data. Despite these limitations, this polarization‐dependent behavior around 1644 cm^−1^ is reproducible and was consistently observed in control experiments. Taken together, the data from both membrane systems support the hypothesis that photoswitching to the *cis* state alters the membrane environment in a manner that promotes a reorientation of LAH4 toward a more inserted state.

In the case of the LUVs at pH 7.0 (Figure 6c), no distinct feature is observed in the amide I region around 1644 cm^−1^, in contrast to the behavior at pH 4.0. The spectral changes present in the pH 7.0 double difference spectra can be explained by small differences in frequencies or bandwidths that give rise to apparent positive and negative signals after repeated subtraction. This reveals the limitations associated with the analysis of weak signals in multiple‐difference spectra. A similar result is observed for the nanodiscs at pH 7.0 (Figure 6d), as expected since the photoinduced spectral changes are even smaller than in LUVs. Consequently, no photoswitching‐induced change in peptide orientation was detected at pH 7.0. If an orientational response does occur, it is likely too small to be resolved within the sensitivity limits of the measurements. Our measurements show that the photoswitching does induce conformational changes in the peptide at both pH values (Figure 5); however, a change in the helix orientation is only observed for the surface‐bound peptide (pH 4.0). At pH 7.0, the peptide is inserted into the membrane and therefore more strongly anchored within the membrane. As a result, the structural perturbations induced by photoswitching appear insufficient to induce a detectable reorientation of the peptide, and its overall orientation remains largely unchanged.

## 4 Conclusion

In this work, the α‐helical model peptide LAH4 was successfully characterized in LUVs and in nanodiscs using polarized ATR‐FTIR spectroscopy. The pH‐dependent orientation of LAH4 could be clearly resolved in both membrane systems. Incorporation of the photoswitchable lipid azoPC enabled light‐induced modulation of membrane properties, with a stronger photoswitching response observed in LUVs than in nanodiscs due to the constraints of the scaffolding copolymer. Unpolarized measurements revealed photoswitching‐dependent conformational changes of the peptide at both studied pH values, indicating that membrane dynamics generated by azoPC isomerization affects peptide–membrane interaction. Polarized measurements at pH 4.0 show that the LAH4 α‐helix is oriented largely parallel to the membrane surface in the *trans* state. Upon switching to the *cis* state, the membrane becomes more expanded and “opens up”, promoting the reorientation of the peptide toward a more membrane‐inserted state. This effect was most evident in LUVs, but also observed to a lesser extent in nanodiscs, as expected due to membrane constraints imposed by the surrounding copolymer. In contrast, no detectable photoswitching‐dependent changes in peptide orientation were observed at pH 7.0 in either membrane system. The peptide is inserted into the membrane under these conditions. Minor conformational changes do occur upon photoswitching, but no orientational changes occur since the peptide is more anchored within the lipid bilayer.

Future studies could extend this approach to other antimicrobial peptides in order to assess whether the photoswitching‐induced effects observed for LAH4 represent a more general phenomenon for the interplay of membrane dynamics and peptide insertion. Such investigations would be particularly valuable, as many antimicrobial peptides exert their biological function through membrane binding and insertion, making them highly sensitive to changes in membrane structure and dynamics. Furthermore, investigating nanodiscs of different diameters and consequently different lateral pressure profiles may provide deeper insights into the role of membrane confinement and mechanical membrane properties in modulating peptide–membrane interactions. These approaches would help to disentangle the relative contributions of membrane dynamics, copolymer effects, and peptide insertion depth, and further establish nanodiscs as a versatile platform for light‐controlled membrane studies.

## Funding

This work was supported by the Deutsche Forschungsgemeinschaft (DFG, German Research Foundation) – 564132680 and SFB 1756 – 550938463.

## Conflicts of Interest

The authors declare no conflicts of interest.

## Supporting information

Supplementary Material

## Data Availability

The data that support the findings of this study are available in the Supporting Information of this article.

## References

[1] H. Watson , “Biological Membranes,” Essays in Biochemistry 59 (2015): 43–69, 10.1042/bse0590043. 26504250 PMC4626904

[2] N. Nisticò , M. Greco , M. C. Bruno , E. Giuliano , P. Sinopoli , and D. Cosco , “Biomimetic Lipid Membranes: An Overview on Their Properties and Applications,” Applied Materials Today 35 (2023): 101998, 10.1016/j.apmt.2023.101998.

[3] T. H. Bayburt , Y. V. Grinkova , and S. G. Sligar , “Self‐Assembly of Discoidal Phospholipid Bilayer Nanoparticles With Membrane Scaffold Proteins,” Nano Letters 2 (2002): 853–856, 10.1021/nl025623k.

[4] T. H. Bayburt and S. G. Sligar , “Membrane Protein Assembly Into Nanodiscs,” FEBS Letters 584 (2010): 1721–1727, 10.1016/j.febslet.2009.10.024. 19836392 PMC4758813

[5] I. G. Denisov , Y. V. Grinkova , A. A. Lazarides , and S. G. Sligar , “Directed Self‐Assembly of Monodisperse Phospholipid Bilayer Nanodiscs With Controlled Size,” Journal of the American Chemical Society 126 (2004): 3477–3487, 10.1021/ja0393574. 15025475

[6] A. O. Oluwole , J. Klingler , B. Danielczak , et al., “Formation of Lipid‐Bilayer Nanodiscs by Diisobutylene/Maleic Acid (DIBMA) Copolymer,” Langmuir 33 (2017): 14378–14388, 10.1021/acs.langmuir.7b03742. 29160078

[7] A. O. Oluwole , B. Danielczak , A. Meister , J. O. Babalola , C. Vargas , and S. Keller , “Solubilization of Membrane Proteins Into Functional Lipid‐Bilayer Nanodiscs Using a Diisobutylene/Maleic Acid Copolymer,” Angewandte Chemie International Edition 56 (2017): 1919–1924, 10.1002/anie.201610778. 28079955 PMC5299484

[8] I. G. Denisov and S. G. Sligar , “Nanodiscs for Structural and Functional Studies of Membrane Proteins,” Nature Structural & Molecular Biology 23 (2016): 481–486, 10.1038/nsmb.3195. PMC8934039 27273631

[9] S. T. Henriques , M. N. Melo , and M. A. Castanho , “Cell‐Penetrating Peptides and Antimicrobial Peptides: How Different Are They?,” Biochemical Journal 399 (2006): 1–7, 10.1042/BJ20061100. 16956326 PMC1570158

[10] M. Zasloff , “Antimicrobial Peptides of Multicellular Organisms,” Nature 415 (2002): 389–395. 11807545 10.1038/415389a

[11] J. Morstein , A. C. Impastato , and D. Trauner , “Photoswitchable Lipids,” Chembiochem 22 (2021): 73–83, 10.1002/cbic.202000449. 32790211

[12] K. Mors , C. Roos , F. Scholz , et al., “Modified Lipid and Protein Dynamics in Nanodiscs,” Biochimica et Biophysica Acta (BBA)—Biomembranes 1828 (2013): 1222–1229, 10.1016/j.bbamem.2012.12.011. 23276833

[13] C. Pernpeintner , J. A. Frank , P. Urban , et al., “Light‐Controlled Membrane Mechanics and Shape Transitions of Photoswitchable Lipid Vesicles,” Langmuir 33 (2017): 4083–4089, 10.1021/acs.langmuir.7b01020. 28361538

[14] L. Socrier and C. Steinem , “Photo‐Lipids: Light‐Sensitive Nano‐Switches to Control Membrane Properties,” ChemPlusChem 88 (2023): e202300203, 10.1002/cplu.202300203. 37395458

[15] S. D. Pritzl , J. Morstein , N. A. Pritzl , J. Lipfert , T. Lohmuller , and D. H. Trauner , “Photoswitchable Phospholipids for the Optical Control of Membrane Processes, Protein Function, and Drug Delivery,” Communications Materials 6 (2025): 59, 10.1038/s43246-025-00773-8. 40182703 PMC11961368

[16] V. Marturano , V. Ambrogi , N. A. G. Bandeira , B. Tylkowski , M. Giamberini , and P. Cerruti , “Modeling of Azobenzene‐Based Compounds,” Physical Sciences Reviews 2 (2017): 20170138, 10.1515/psr-2017-0138.

[17] P. Urban , S. D. Pritzl , M. F. Ober , et al., “A Lipid Photoswitch Controls Fluidity in Supported Bilayer Membranes,” Langmuir 36 (2020): 2629–2634, 10.1021/acs.langmuir.9b02942. 32069411

[18] X. Song , J. Perlstein , and D. G. Whitten , “Supramolecular Aggregates of Azobenzene Phospholipids and Related Compounds in Bilayer Assemblies and Other Microheterogeneous Media: Structure, Properties, and Photoreactivity1,” Journal of the American Chemical Society 119 (1997): 9144–9159, 10.1021/ja971291n.

[19] P. Urban , S. D. Pritzl , D. B. Konrad , et al., “Light‐Controlled Lipid Interaction and Membrane Organization in Photolipid Bilayer Vesicles,” Langmuir 34 (2018): 13368–13374, 10.1021/acs.langmuir.8b03241. 30346771

[20] M. Doroudgar , J. Morstein , J. Becker‐Baldus , D. Trauner , and C. Glaubitz , “How Photoswitchable Lipids Affect the Order and Dynamics of Lipid Bilayers and Embedded Proteins,” Journal of the American Chemical Society 143 (2021): 9515–9528, 10.1021/jacs.1c03524. 34133158

[21] B. Bechinger , “Toward Membrane Protein Design‐ pH‐Sensitive Topology of Histidine‐Containing Polypeptides,” Journal of Molecular Biology 263 (1996): 768–775, 10.1006/jmbi.1996.0614. 8947574

[22] H. G. Boman , “Antibacterial Peptides: Basic Facts and Emerging Concepts,” Journal of Internal Medicine 254 (2003): 197–215, 10.1046/j.1365-2796.2003.01228.x. 12930229

[23] M. Sugawara , J. M. Resende , C. M. Moraes , et al., “Membrane Structure and Interactions of Human Catestatin by Multidimensional Solution and Solid‐State NMR Spectroscopy,” FASEB Journal 24 (2010): 1737–1746, 10.1096/fj.09-142554. 20103720

[24] C. Aisenbrey , R. Kinder , E. Goormaghtigh , J. M. Ruysschaert , and B. Bechinger , “Interactions Involved in the Realignment of Membrane‐Associated Helices. An Investigation Using Oriented Solid‐State NMR and Attenuated Total Reflection Fourier Transform Infrared Spectroscopies,” Journal of Biological Chemistry 281 (2006): 7708–7716, 10.1074/jbc.M513151200. 16407268

[25] C. Aisenbrey , E. Goormaghtigh , J. M. Ruysschaert , and B. Bechinger , “Translocation of Amino Acyl Residues From the Membrane Interface to the Hydrophobic Core: Thermodynamic Model and Experimental Analysis Using ATR‐FTIR Spectroscopy,” Molecular Membrane Biology 23 (2010): 363–374, 10.1080/09687860600738742. 16923729

[26] J. Georgescu , V. H. Munhoz , and B. Bechinger , “NMR Structures of the Histidine‐Rich Peptide LAH4 in Micellar Environments: Membrane Insertion, pH‐Dependent Mode of Antimicrobial Action, and DNA Transfection,” Biophysical Journal 99 (2010): 2507–2515, 10.1016/j.bpj.2010.05.038. 20959091 PMC2955508

[27] B. Perrone , A. J. Miles , E. S. Salnikov , B. A. Wallace , and B. Bechinger , “Lipid Interactions of LAH4, a Peptide With Antimicrobial and Nucleic Acid Transfection Activities,” European Biophysics Journal 43 (2014): 499–507, 10.1007/s00249-014-0980-y. 25182242

[28] B. Bechinger , J. M. Ruysschaert , and E. Goormaghtigh , “Membrane Helix Orientation From Linear Dichroism of Infrared Attenuated Total Reflection Spectra,” Biophysical Journal 76 (1999): 552–563, 10.1016/S0006-3495(99)77223-1. 9876168 PMC1302545

[29] J. Wolf , C. Aisenbrey , N. Harmouche , et al., “pH‐Dependent Membrane Interactions of the Histidine‐Rich Cell‐Penetrating Peptide LAH4‐L1,” Biophysical Journal 113 (2017): 1290–1300, 10.1016/j.bpj.2017.06.053. 28734478 PMC5607044

[30] E. Goormaghtigh , V. Raussens , and J. M. Ruysschaert , “Attenuated Total Reflection Infrared Spectroscopy of Proteins and Lipids in Biological Membranes,” Biochimica et Biophysica Acta 1999 (1422): 105–185, 10.1016/S0304-4157(99)00004-0. 10393271

[31] M. A. Fallah , H. R. Gerding , C. Scheibe , et al., “Simultaneous IR‐Spectroscopic Observation of Alpha‐Synuclein, Lipids, and Solvent Reveals an Alternative Membrane‐Induced Oligomerization Pathway,” ChemBioChem 18 (2017): 2312–2316, 10.1002/cbic.201700355. 28980756

[32] N. Voievoda , T. Schulthess , B. Bechinger , and J. Seelig , “Thermodynamic and Biophysical Analysis of the Membrane‐Association of a Histidine‐Rich Peptide With Efficient Antimicrobial and Transfection Activities,” Journal of Physical Chemistry B 119 (2015): 9678–9687, 10.1021/acs.jpcb.5b04543. 26134591

[33] A. Farrotti , G. Bocchinfuso , A. Palleschi , et al., “Molecular Dynamics Methods to Predict Peptide Locations in Membranes: LAH4 as a Stringent Test Case,” Biochimica et Biophysica Acta (BBA)—Biomembranes 1848 (2015): 581–592, 10.1016/j.bbamem.2014.11.002. 25445672

[34] L. K. Tamm and S. A. Tatulian , “Infrared Spectroscopy of Proteins and Peptides in Lipid Bilayers,” Quarterly Reviews of Biophysics 30 (1997): 365–429, 10.1017/s0033583597003375. 9634652

[35] F. Crea , A. Vorkas , A. Redlich , et al., “Photoactivation of a Mechanosensitive Channel,” Frontiers in Molecular Biosciences 9 (2022): 905306, 10.3389/fmolb.2022.905306. 35836929 PMC9273776

[36] F. Baserga , A. Vorkas , F. Crea , et al., “Membrane Protein Activity Induces Specific Molecular Changes in Nanodiscs Monitored by FTIR Difference Spectroscopy,” Frontiers in Molecular Biosciences 9 (2022): 915328, 10.3389/fmolb.2022.915328. 35769914 PMC9234331

[37] T. Golz , E. Bau , J. Zhang , et al., “Transient Infrared Nanoscopy Resolves the Millisecond Photoswitching Dynamics of Single Lipid Vesicles in Water,” Nature Communications 16 (2025): 6033, 10.1038/s41467-025-61341-9. PMC12216292 40593745

[38] C. Kammerer , D. Trauner , and T. Lohmueller , “Photolipid Isomerization in Multicomponent Lipid Membranes: Effects of Temperature and Charge,” Langmuir 41 (2025): 23025–23035, 10.1021/acs.langmuir.5c02698. 40833068

[39] M. Aleksanyan , A. Grafmüller , F. Crea , et al., “Photomanipulation of Minimal Synthetic Cells: Area Increase, Softening, and Interleaflet Coupling of Membrane Models Doped With Azobenzene‐Lipid Photoswitches,” Advanced Science 10 (2023): 2198–3844, 10.1002/advs.202304336. PMC10625111 37653602

