# Supplementary material for: Polarized ATR‐FTIR Spectroscopy Reveals Light‐Induced Peptide Insertion of LAH4 Into Photoswitchable Membranes
Source: Chemphyschem. 2026 Aug 31;27(17):e70554. doi: 10.1002/cphc.70554 (PMC13530310; doi:10.1002/cphc.70554)
Supplement: Supplementary file 1 [file CPHC-27-e70554-s001.pdf]

SUPPORTING INFORMATION

Polarized ATR-FTIR Spectroscopy Reveals Light-Induced Peptide Insertion of LAH4 into Photoswitchable Membranes

Celine Eckert, Selina Mertens and Karin Hauser*

Department of Chemistry, University of Konstanz, 78457 Konstanz, Germany

* Correspondence: karin.hauser@uni-konstanz.de

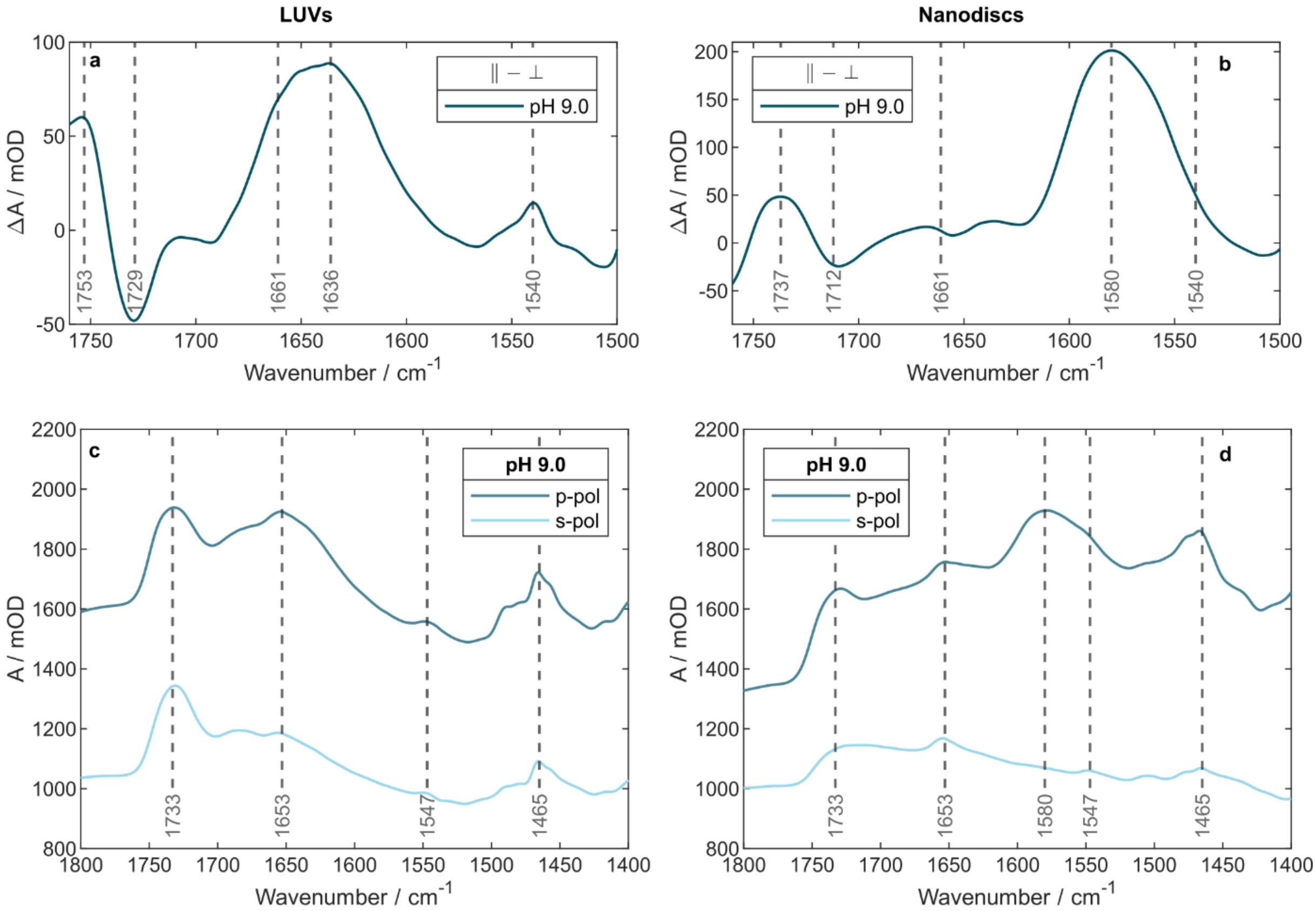

Figure S1 Polarized ATR-FTIR spectra of POPC LUVs (a,c) and DIBMA-stabilized nanodiscs (b,d) with the peptide LAH4 at pH 9.0. The absorption spectra recorded with parallel (p-pol) and perpendicular (s-pol) polarization are shown for LUVs (c) and nanodiscs (d). In general, both membrane systems exhibit similar spectral features, with the notable exception of a broad band around 1580 cm^{-1} observed in the nanodiscs spectra, particularly in the p-polarized measurement. This band is absent in the corresponding LUVs spectra and is attributed to the deprotonated carboxylate groups (COO^-) of the DIBMA copolymer, which becomes populated under basic conditions. To determine the orientation of LAH4, dichroic spectra were generated by subtracting the s-polarized spectra from the corresponding p-polarized spectra (with a factor of 1.34). As shown for the LUVs (a), the expected positive band in the amide I region associated with an inserted peptide orientation is observed. In contrast, the dichroic spectrum of the nanodiscs (b) is dominated by the broad COO^- band at 1580 cm^{-1} and thus overlays weaker peptide-related signals in the amide I and amide II regions. Consequently, the interpretation of peptide orientation in nanodiscs at pH 9.0 is complicated by spectral overlap arising from the copolymer. For this reason, pH 7.0 was selected for all measurements to minimize interference from the DIBMA carboxylate vibration while maintaining conditions that favor membrane insertion of LAH4.

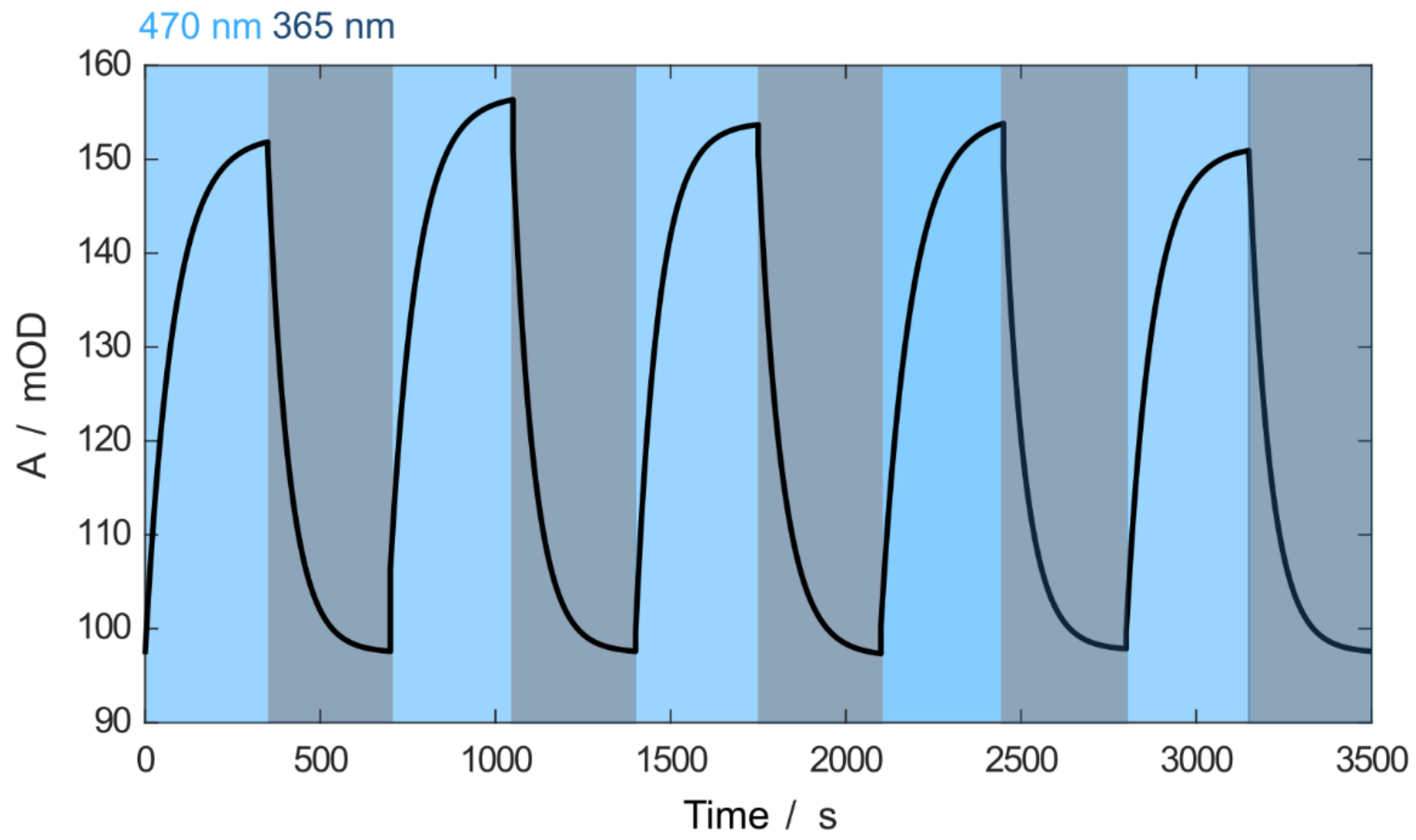

Figure S2. Rapid-scan ATR-FTIR difference spectra of LUVs composed of 50 % POPC and 50 % azoPC (Tris-HCl buffer). The photoswitching reversibility from the absorbance changes at 1603 cm^{-1} is shown for five cycles with monoexponential fits for the recorded transients, corresponding to alternating illumination with blue light at 470 nm (3.24 mW cm^{-2}) and UV light at 365 nm (15.59 mW cm^{-2}) for 350 s each.

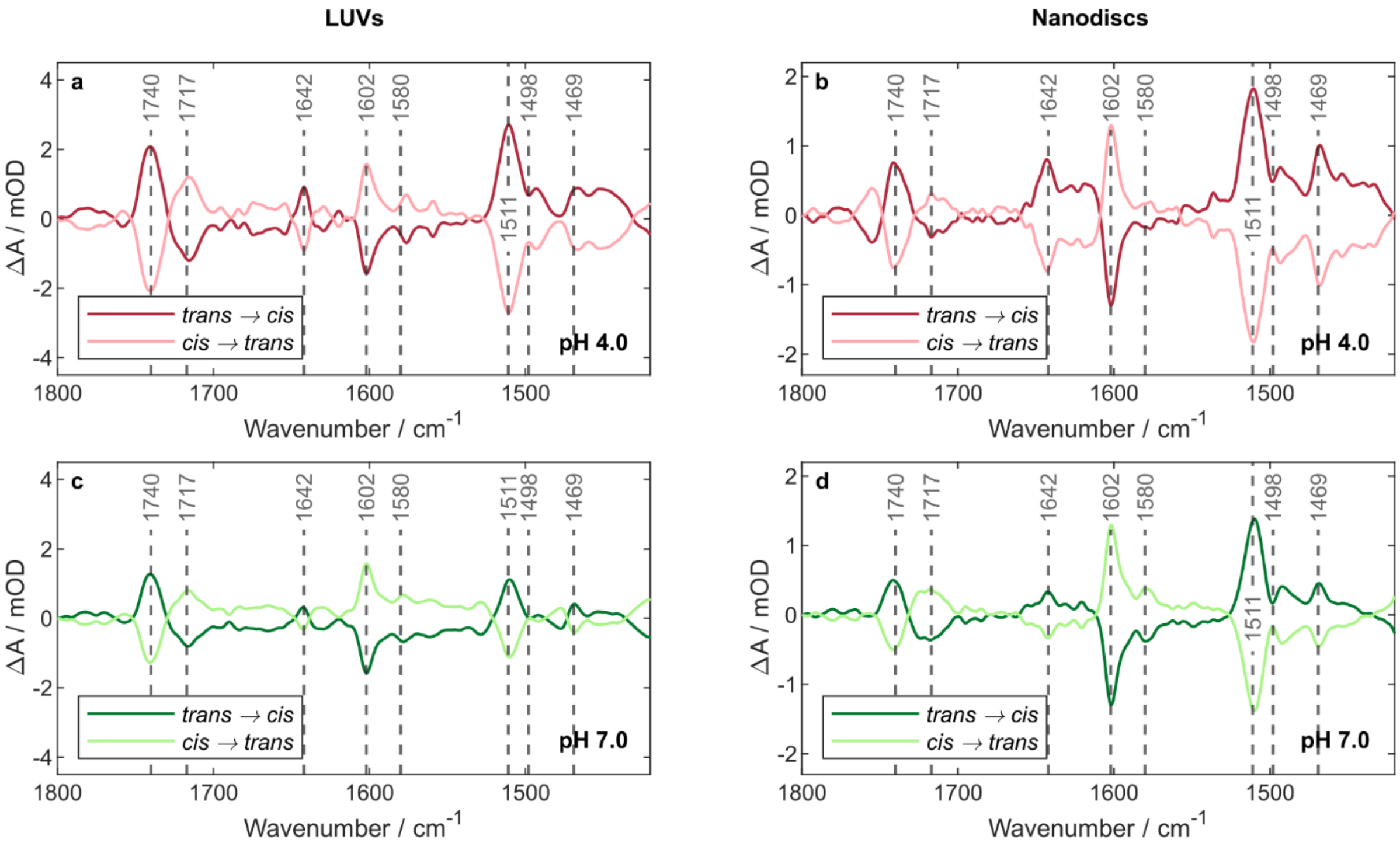

Figure S3. ATR-FTIR difference spectra of LUVs (a,c) and nanodiscs (b,d) composed of 50 % POPC and 50 % azoPC with the peptide LAH4 at pH 4.0 (red, a,b) and pH 7.0 (green, c,d). The spectra illustrate the photoinduced transition of azoPC from *trans*-to-*cis* isomer (dark colored) and from *cis*-to-*trans* isomer (light colored). Structural changes of the membrane photoswitching are reflected in the characteristic azoPC marker bands (like in Figure 3, main text). In addition, a band at 1642 cm^{-1} appears in the amide I region, indicating the presence of photoinduced conformational peptide changes. This is observed in both membrane systems and at both pH values. Notably, the difference spectra exhibit a positive amide I band during the *trans*-to-*cis* isomerization, whereas the corresponding isomerization back to the *trans* state results in a negative band. This mirror-image relationship further supports the interpretation that the peptide response is directly linked to the reversible structural changes induced by the membrane photoswitching.
